# Femoral Plaque Burden and Left Ventricular–Arterial Coupling in Patients with Chronic Heart Failure

**DOI:** 10.3390/jcm15052014

**Published:** 2026-03-06

**Authors:** Vadim Genkel, Sergey Ershov, Evgeny Lebedev, Yana Zaripova, Igor Shaposhnik

**Affiliations:** 1 Department of Internal Medicine, South Ural State Medical University, Chelyabinsk 454141, Russia; evgueni.lebedev@mail.ru (E.L.); yanakud123@mail.ru (Y.Z.); shaposhnik@yandex.ru (I.S.); 2 City Clinical Hospital No. 1, Chelyabinsk 454048, Russia; sergei.eee-ershov@yandex.ru

**Keywords:** chronic heart failure, femoral plaque burden, left ventricular function, left atrial function, ventricular–arterial coupling

## Abstract

**Background/Objectives:** Lower extremity peripheral artery disease (PAD) is recognized as a significant public health issue, particularly due to its strong association with adverse cardiovascular events. Despite this, little attention has been given to its influence on left ventricular (LV) and left atrial (LA) function in patients with chronic heart failure (CHF). This study aims to examine the relationship between femoral plaque burden and structural and functional properties of the LV and LA in patients with CHF. **Methods:** Study design: cross-sectional observational single-center study. A total of 89 patients with CHF underwent comprehensive assessments, including duplex ultrasonography of lower extremity arteries and two-dimensional echocardiography. Analysis focused on evaluating femoral plaque burden, left ventricular deformation, and ventricular–arterial coupling. **Results:** Findings indicated that increased femoral plaque burden was associated with reductions in LA deformation and increases in LA stiffness. Similarly, there was evidence of impaired LV mechanics and elevated arterial loading, suggesting impaired ventricular–arterial coupling in patients with CHF and significant lower extremity atherosclerosis. **Conclusions:** Femoral plaque burden is closely linked to detrimental changes in LA and LV function, as well as disturbances in ventricular–arterial coupling, underscoring the importance of addressing lower extremity atherosclerosis in managing CHF patients.

## 1. Introduction

Lower extremity peripheral artery disease (PAD) represents a significant public health challenge in the 21st century, whose global burden has been steadily increasing worldwide for the past 30 years [1]. However, despite the substantial contribution of PAD to reduced quality and length of life, it remains an understudied, under-recognized, underdiagnosed, and undertreated condition compared to atherosclerosis in other vascular territories [2,3]. Furthermore, even early atherosclerosis of the lower extremity arteries and asymptomatic PAD are associated with generalized polyvascular atherosclerosis and a high risk of a broad spectrum of adverse cardiovascular events [4,5,6]. One of the underestimated consequences of atherosclerosis of the lower extremity arteries is likely left ventricular (LV) dysfunction, both in asymptomatic patients and in patients with established chronic heart failure (CHF).

To date, data on the prevalence of PAD in patients with CHF are limited. According to the PINNACLE (Practice Innovation and Clinical Excellence) registry, which included 697,542 patients with CHF as of 2019, the overall prevalence of PAD was 12.9% [7]. In a small clinical study conducted in Brazil involving 106 patients with CHF, PAD was diagnosed in 19.4% of patients [8]. Results from several prospective studies indicate that PAD is associated with an increased relative risk (RR) of both new-onset CHF and decompensation of pre-existing CHF. For instance, an analysis of the MESA (The Multi-Ethnic Study of Atherosclerosis), which included 6553 patients without established cardiovascular disease or heart failure, found that an ankle-brachial index (ABI) < 0.9 at the initial visit was associated with a 2.02-fold increased risk (RR 2.02; 95% CI 1.19–3.40; *p* = 0.01) of developing heart failure with reduced left ventricular ejection fraction (HFrEF) over 14 years of follow-up [9]. Patients with CHF and PAD enrolled in the EMPEROR-Pooled (Empagliflozin Outcome Trial in Patients With Chronic Heart Failure) analysis had a higher risk of hospitalization for heart failure compared to those without PAD (RR 1.51; 95% CI 1.12–2.03; *p* = 0.007) [10].

It should be noted that, according to data from the IMPACT-ABI (impressive predictive value of ankle brachial index for clinical long-term outcome in patients with cardiovascular disease examined by ABI) study, not only an ABI < 0.9 but also borderline ABI values in the range of 0.91 to 0.99 may be associated with an increased risk of CHF (RR 2.68; 95% CI 1.35–5.34; *p* = 0.005) [11]. Moreover, it is hypothesized that even non-stenotic, extensive atherosclerosis of the lower extremity arteries impairs left ventricular–arterial coupling and left atrioventricular coupling (or left atrial coupling index). This, in turn, worsens left ventricular and left atrial function and complicates the course of CHF. However, to date, clinical research data on this subject remain limited. The aim of this study was to investigate the associations of atherosclerosis of lower extremity arteries, including measures of femoral plaque burden, with the structural and functional characteristics of the left ventricle and left atrium.

## 2. Materials and Methods

The study included patients with an established diagnosis of CHF, aged 40 to 79 years, who were under ambulatory and/or inpatient management at the City Clinical Hospital No. 1, Chelyabinsk. Patients were recruited between 9 November 2024 and 27 August 2025. The diagnosis of CHF was established in accordance with current clinical guidelines [12,13]. Patients were excluded from the study if they met any of the following criteria: hemodynamically significant congenital or acquired heart defects, myocarditis, cardiomyopathies, severe liver or kidney dysfunction, chronic viral hepatitis, or HIV infection. The study protocol was approved by the Local Ethics Committee of the South Ural State Medical University (Protocol No. 7, dated 5 November 2024). Upon inclusion in the study, all patients provided written informed consent. Study design: cross-sectional observational single-center study.

### 2.1. Laboratory Study

The laboratory investigation included the measurement of NT-proBNP (“Vector-Best”, Novosibirsk, Russia), total cholesterol, low-density lipoprotein cholesterol (LDL-C), high-density lipoprotein cholesterol (HDL-C), triglycerides, high-sensitivity C-reactive protein (hsCRP; “Vector-Best”, Novosibirsk, Russia), glucose, and creatinine, with subsequent calculation of the glomerular filtration rate using the CKD-EPI formula.

### 2.2. Duplex Ultrasound Scanning of the Lower Extremity Arteries

All patients underwent duplex ultrasound scanning of the lower extremity arteries using a Samsung Medison EKO7 (Samsung Medison Co., Seoul, Republic of Korea) digital ultrasound system with a 10 MHz linear transducer. The examination was performed in B-mode, color Doppler mapping, and pulsed-wave Doppler modes. The following vessels were examined bilaterally in longitudinal and transverse sections along their entire length: the common femoral arteries (CFA), superficial femoral arteries (SFA), popliteal arteries (PA), tibioperoneal trunk, anterior tibial arteries (ATA), and posterior tibial arteries (PTA). An atherosclerotic plaque was defined as a focal thickening of the intima-media complex exceeding 1.5 mm, being more than 0.5 mm thicker than the surrounding intima-media thickness (IMT), or exceeding the IMT of adjacent segments by more than 50% [14]. The percentage of stenosis was measured planimetrically in B-mode based on the vessel diameter in the transverse cross-section, according to the ECST (The European Carotid Surgery Trial) method.

**Figure 1:**
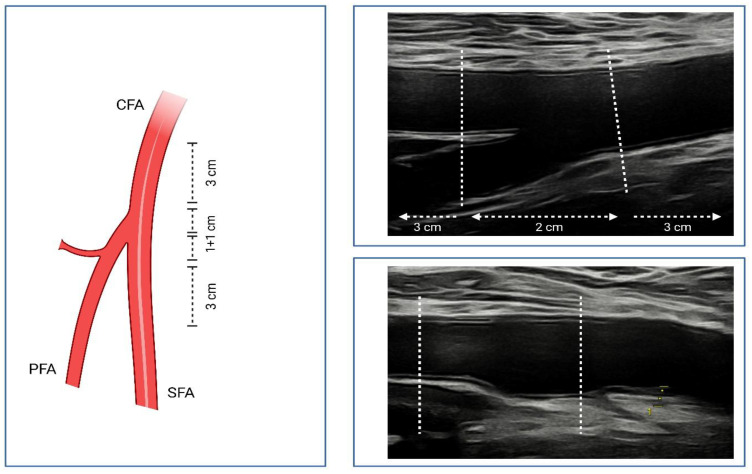
Methodology of ultrasound examination of the femoral arteries. The division of the femoral artery bifurcation into segments and an example of the division of the femoral artery bifurcation during duplex scanning are shown. The bottom right image shows the measurement of the femoral plaque thickness in segment S3. Created in BioRender. Genkel, V. (2026) https://BioRender.com/t4js8m9. CFA: common femoral artery; PFA: profunda femoris artery; SFA: superficial femoral artery.

To assess the femoral plaque burden, the distal part of CFA, the CFA bifurcation, and the proximal segments of the superficial femoral artery (SFA) and profunda femoris artery (PFA) were divided into the following segments: S3—distal part of CFA (3 cm segment), S2—CFA bifurcation, S1—proximal parts of the SFA and PFA (3 cm segments) [5]. To determine the plaque area, the optimal longitudinal view for visualizing each atherosclerotic plaque was obtained, and the perimeter of every plaque within the examined femoral artery segments was manually traced. The femoral total plaque area (fTPA) was defined as the sum of all plaque areas from both sides [5,15]. The femoral total plaque thickness (fTPT) was calculated by summing the maximum plaque thickness measured at the near and far walls of the three segments from both femoral arteries (see Figure 1) [16].

FTPA, fTPT and femoral total plaque number (fTPN) were measured along three segments of the femoral artery. The maximum percentage of stenosis (MaxStenosis) was defined as the highest stenosis in the examined segments of the femoral arteries on both sides.

All measurements were performed by a single trained operator. The intra-operator reproducibility was determined for the measured indicators. For this purpose, measurements were taken in 11 patients at 24–48 h intervals, followed by a calculation of the coefficient of intra-class correlation with 95% confidence interval (CI): fTPA—0.998 (95% CI 0.992–0.999); fTPT—0.998 (95% CI 0.992–0.999); MaxStenosis—0.979 (95% CI 0.923–0.994).

The ABI was measured using pulsed-wave Doppler by a Samsung Medison EKO7 (Samsung Medison Co., Seoul, Republic of Korea) digital ultrasound system with a 10 MHz linear transducer. The numerator was defined as the highest systolic blood pressure recorded in the target lower limb from the posterior tibial artery and the dorsalis pedis artery. The denominator was defined as the highest systolic blood pressure recorded from either upper limb. The mean ABI (ABIm) was calculated as the arithmetic mean between the right and left ABI. ABI measurement was performed in accordance with the 2024 ESC Guidelines for the management of peripheral arterial and aortic diseases [17].

### 2.3. Echocardiography

Two-dimensional echocardiography was performed in accordance with the guidelines of the European Association of Cardiovascular Imaging (EACVI), the American Society of Echocardiography (ASE), and the Russian Society of Cardiology [18,19,20]. To evaluate systolic function, advanced echocardiography was performed: a 2D speckle-tracking study with assessment of left ventricular global longitudinal strain (LVGLS), left ventricular circumferential strain (LVCS), and strain rate (SR). Left ventricular ejection fraction (LVEF) was measured using the biplane Simpson’s method. Left atrial global longitudinal strain was also assessed, with separate analysis of the left atrial reservoir phase strain (LASr). In patients with atrial fibrillation, left atrial and left ventricular strain measurements were indexed with the square root of the RR-interval measured during the same heart cycle as strain was obtained [21]. The measurement methodology for the investigated parameters is presented in Table 1.

Abnormal elevation of Ea/Ees, reflecting ventricular–arterial decoupling, was defined as an increase in Ea/Ees ≥ 1. Although Ea/Ees can be in a wide range of values in a physiological state, an increase in Ea/Ees ≥ 1 in patients with HF is most often considered an indicator of ventricular–arterial coupling mismatch [32,33,34].

All measurements were performed by a single trained operator. The intra-operator reproducibility was determined for the directly measured indicators. For this purpose, measurements were taken in 11 patients at 24–48 h intervals, followed by a calculation of the coefficient of intra-class correlation with 95% CI: IVSd—0.947 (95% CI 0.815–0.985); PWT—0.954 (95% CI 0.840–0.987); LVEDV—0.988 (95% CI 0.957–0.997); LVESV—0.994 (95% CI 0.976–0.998); LVOT—0.947 (95% CI 0.820–0.985); VTI LVOT—0.968 (95% CI 0.887–0.991); LAV—0.985 (95% CI 0.945–0.996); LAGLS—0.966 (95% CI 0.879–0.991); LASr—0.952 (95% CI 0.834–0.987); LVGLS—0.887 (95% CI 0.636–0.968); LVGLSR—0.830 (95% CI 0.486–0.951); LVCS—0.980 (95% CI 0.930–0.994).

### 2.4. Statistical Analysis

The statistical analysis of the obtained data was performed using MedCalc software (version 23.3.7, MedCalc Software Ltd., Osten, Belgium) and OriginPro 2024 (OriginLab Co., Northampton, MA, USA). Qualitative variables are described using absolute and relative frequencies (percentages). Quantitative variables are described by the median (Me) with the interquartile range specified [25th percentile; 75th percentile]. The Mann–Whitney U test was used to assess the significance of differences between two groups. The Chi-square test was used to assess the significance of differences in the frequency distribution of nominal variables between two groups. Spearman’s correlation analysis was used to determine relationships between parameters. Logistic regression analysis was used to identify independent predictors of the dependent variable, allowing for the assessment of the relationship between a binary categorical variable and one or several other variables, both continuous and categorical. To identify dose–response relationships, a dose–response analysis (probit regression analysis) was performed. To establish threshold values for the studied parameters, ROC analysis was performed with determination of sensitivity and specificity, as well as calculation of the area under the characteristic curve (AUC) with a 95% confidence interval (CI). Statistical significance was considered at a critical level of 0.05.

A preliminary calculation of the required sample size for the planned study was performed, taking into account the testing hypotheses and proposed statistical procedures [35]. To identify potential associations between echocardiographic parameters and indicators of femoral plaque burden using correlation analysis, the minimum sample size for a correct identification of associations with a correlation coefficient ≥ 0.300 was 84 participants (type I error 0.05; type II error 0.20) [36]. For identifying the potential diagnostic accuracy (moderate-to-high with an AUC ≥ 0.700) of femoral plaque burden indicators in relation to detecting ventricular–arterial decoupling using ROC analysis, the minimum required sample size was 72 participants (type I error 0.05; type II error 0.20; ratio of sample sizes in negative/positive groups 2.0) [37]. Therefore, the minimally required sample size was defined as more than 85 participants.

## 3. Results

The study enrolled 89 patients with CHF. The clinical and laboratory characteristics of the patients are presented in Table 2.

As shown in Table 2, the study sample predominantly consisted of patients with HFpEF NYHA class III-IV. It is noteworthy that the medical therapy presented reflects the treatment regimen at the time of study enrollment. The results of the duplex ultrasound scanning of the lower extremity arteries and the echocardiography are presented in Table 3.

Thus, the vast majority of patients presented with atherosclerotic plaques in the lower extremity arteries. However, a reduction in ABI < 0.9 was diagnosed in only 7.86% of patients, and the median degree of stenosis was approximately 30%. Left ventricular systolic dysfunction, assessed by GLS, was detected in 85.4% of patients, whereas a reduced LVEF was observed in only 29.2%.

**Figure 2:**
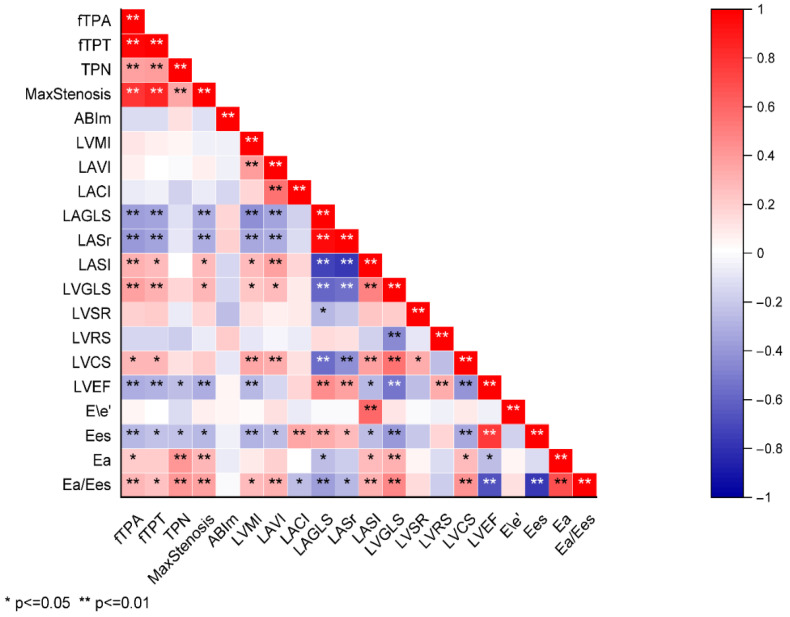
Heat map of correlations between indicators of femoral plaque burden and echocardiographic parameters.

To identify the relationships between the ultrasound-based markers of lower extremity artery atherosclerosis burden and the echocardiographic parameters, a correlation analysis was performed. The results are presented in Figure 2.

An increased femoral plaque burden and the degree of lower extremity arterial stenosis were associated with impairments in the structure and function of both the LV and LA. Specifically, higher fTPA, fTPT, and degree of arterial stenosis were linked to an increase in the LASI and a decrease in LAGLS and LASr. Furthermore, greater severity of lower extremity atherosclerosis was associated with reduced longitudinal and circumferential LV strain and lower LVEF. An increased femoral plaque burden was also associated with elevated indices of left ventricular–arterial coupling (Ea/Ees), but not with indices of atrioventricular coupling (LACI).

**Figure 3:**
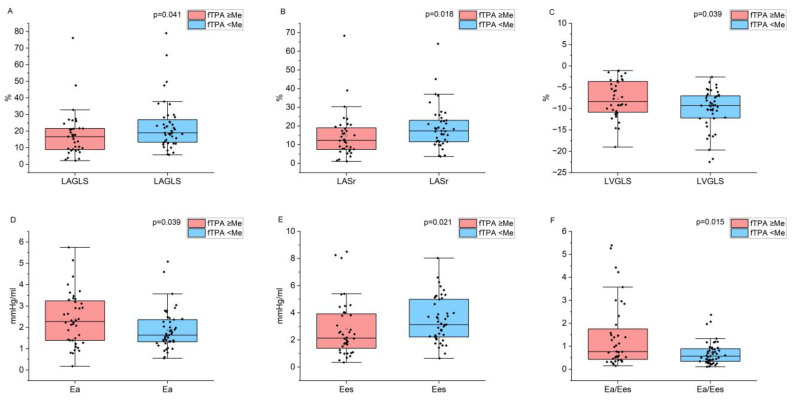
Indicators of LA and LV strain, and arterial and LV elastance depending on the femoral plaque burden: (**A**) LAGLS; (**B**) LASr; (**C**) LVGLS; (**D**) arterial elastance (Ea); (**E**) ventricular elastance (Ees); (**F**) left ventricular–arterial coupling (Ea/Ees).

Subsequently, differences in echocardiographic parameters were analyzed based on the severity of femoral plaque burden. Patients with fTPA values above the median had significantly lower LAGLS, LASr, and LV elastance (Ees), along with higher values for LV GLS, arterial elastance (Ea), and left ventricular–arterial coupling (see Figure 3).

**Figure 4:**
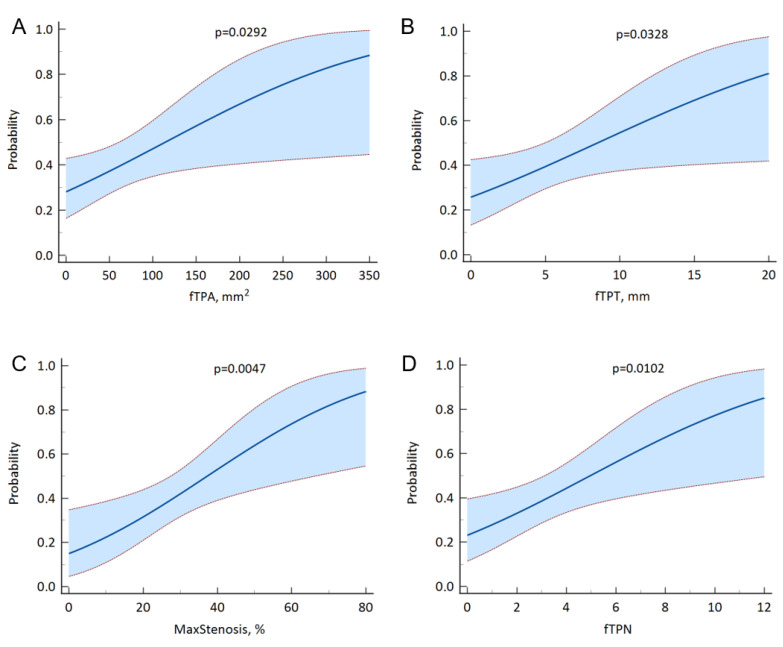
Dose–response relationship between fTPA (**A**), fTPT (**B**), MaxStenosis (**C**), and TPN (**D**), and increase in Ea/Ees ≥ 1.0.

In order to identify dose–response relationships between the studied indicators of atherosclerosis of the lower extremity arteries and an increase in Ea/Ees, a dose–response analysis was performed, the results of which are presented in Figure 4.

**Figure 5:**
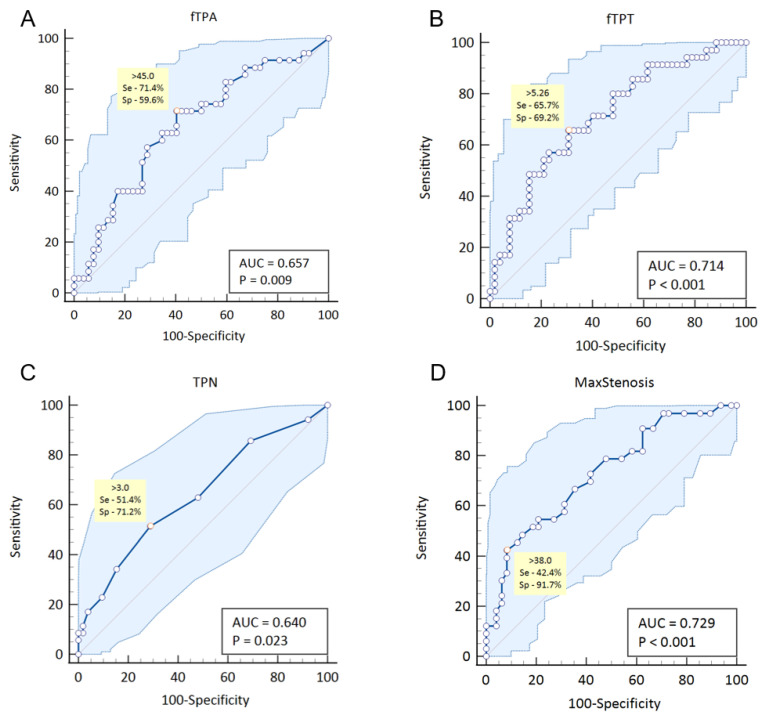
Diagnostic efficacy of fTPA (**A**), fTPT (**B**), TPN (**C**), and MaxStenosis (**D**) in relation to increased left ventricular–arterial coupling (Ea/Ees ≥ 1.0).

Assessment of the diagnostic performance of various ultrasound indicators of femoral plaque burden in detecting elevated left ventricular–arterial coupling (Ea/Ees ≥ 1.0) revealed a moderate to high performance for fTPA, TPN, fTPT, and MaxStenosis (see Figure 5). In contrast, the ABI showed no diagnostic value for identifying a pathological increase in left ventricular–arterial coupling (AUC 0.521; *p* = 0.777).

In order to establish an independent relationship between the indicators of femoral atherosclerosis and the increase in Ea/Ees ≥ 1.0, a logistic regression analysis was performed, the results of which are presented in Table 4.

Therefore, only MaxStenosis of the femoral artery was associated with an increase in the odds ratio of Ea/Ees ≥ 1.0, independently of LVEF, comorbidities, and therapy.

## 4. Discussion

It is well established that lower extremity PAD is associated with an increased risk of adverse limb events, major adverse cardiovascular events, and is a cause of reduced quality of life, walking performance and functional status [38]. In recent years, the potential effects of lower extremity PAD and early atherosclerosis of peripheral arteries on LA and LV mechanical dysfunction, and the associated CHF and cardiac arrhythmias, have been actively investigated [39]. However, data on the relationships between the femoral plaque burden in patients with asymptomatic atherosclerosis of peripheral arteries and parameters of LA and LV mechanical function in patients with CHF are currently lacking. The main results of the present study are as follows: (1) in patients with CHF, an increased femoral plaque burden was associated with reduced deformation and increased stiffness of the LA, as well as reduced deformation and ejection fraction of the LV; (2) in patients with CHF, an increased femoral plaque burden was associated with increased Ea, decreased Ees, and an elevated Ea/Ees ratio; (3) ultrasound indicators of femoral plaque burden demonstrated moderate diagnostic performance in identifying a pathologically elevated Ea/Ees ratio; (4) MaxStenosis of the femoral artery was associated with an increase in the odds ratio of Ea/Ees ≥ 1.0, independently of LVEF, comorbidities, and therapy.

Several previous studies have examined the impact of lower extremity PAD on LV systolic and diastolic function. In the study by K. Yanaka et al., patients with lower extremity PAD defined by a reduced ABI were found to have higher E/e′, tricuspid regurgitation velocity, and left atrial volume index compared to patients without lower extremity PAD [40]. Furthermore, the presence of lower extremity PAD was an independent predictor of LV diastolic dysfunction (OR 1.77; 95% CI 1.13–2.65; *p* = 0.01). In another study by Y.H. Lin et al., pathological ABI values (≤0.90 or >1.4) were an independent predictor of preclinical LV systolic dysfunction (LV GLS ≥ −18%) in multivariate analysis [41]. Moreover, a decreased ABI was associated with increased E/e′ and impaired longitudinal and circumferential LV strain. Our study is the first to demonstrate that the femoral plaque burden in patients with CHF is associated with impaired deformation of not only the LV (GLS and CS) but also the LA (GLS and LASr). Additionally, an increased femoral plaque burden was associated with increased LA stiffness. Various mechanisms linking lower extremity PAD to LV and LA dysfunction are currently under consideration, including endothelial dysfunction and chronic inflammation [42,43]. However, impaired ventricular–arterial coupling is likely one of the primary mechanisms [44]. In the present study, the femoral plaque burden directly correlated with Ea and inversely correlated with Ees, which in turn was associated with an increased Ea/Ees ratio.

An increase in Ea, or effective arterial elastance, is traditionally viewed primarily as a derivative of increased aortic stiffness [45,46]. However, in patients with extensive lower extremity artery atherosclerosis, its contribution to systemic vascular resistance may be significant, and the femoral plaque burden may be an important determinant of Ea [46,47,48]. On the other hand, increased pulse wave velocity associated with extensive atherosclerosis, along with its early reflection and return to the heart during late systole instead of diastole, contributes to elevated central arterial pressure, increased LV afterload, LV hypertrophy and fibrosis, and heightened myocardial oxygen demand [49,50]. Concurrently, reduced central diastolic pressure decreases coronary perfusion and promotes myocardial ischemia. All these mechanisms can contribute to diminished LV myocardial contractility and a reduction in Ees [51]. However, further studies assessing both the burden of atherosclerosis and vascular stiffness are needed to evaluate the contribution of generalized atherosclerosis to increased aortic stiffness.

An elevated Ea/Ees ratio in patients with CHF and femoral atherosclerosis indicates impaired ventricular–arterial coupling and inefficient LV performance [52,53]. In turn, impaired ventricular–arterial coupling in patients with CHF and extensive atherosclerosis may be one of the key factors worsening the CHF course in this patient population [54]. For instance, in the study by Z. Ye et al., which included 1308 patients with lower extremity PAD, an increased Ea/Ees ratio was associated with a higher risk of all-cause mortality over a 3.4-year follow-up [55]. In another study by H. Yokoyama et al., involving patients who underwent TAVI, the risk of a composite endpoint including cardiovascular death and heart failure hospitalization increased with higher Ea/Ees values [52]. Increased LV afterload, along with the high cumulative burden of traditional cardiovascular risk factors characterizing patients with high femoral plaque burden, may also promote LA remodeling [51,56]. The present study is the first to demonstrate that both an increase in femoral plaque burden indicators and the Ea/Ees ratio are associated with increased LA stiffness and reduced LA deformation.

It should be noted that patients with HFpEF and HFrEF have different systemic hemodynamic profiles and different characteristics of ventricular–arterial coupling impairment [46]. While in patients with HFrEF, a decrease in Ees and an increase in Ea lead to a corresponding increase in Ea/Ees, in patients with HFpEF, there is an increase in both Ea and Ees, which in turn is associated with a long-term maintenance of Ea/Ees at normal levels [46,57]. However, hypertension and advanced atherosclerosis may be drivers of accelerated Ea increases, which would lead to an elevation of Ea/Ees [58]. Although in most studies patients with HFrEF had significantly higher Ea/Ees values than HFpEF patients, in some studies the Ea/Ees values in these groups were comparable, while in others an increase in Ea/Ees > 1 was observed in both groups [59]. It can be hypothesized that an increase in Ea/Ees, representing inefficient myocardial performance, may serve as a negative prognostic factor across a wide range of LVEF [52].

Recent years have seen active research aimed at identifying various endophenotypes and phenotypic clusters of CHF [60,61]. A number of studies have established phenogroups characterized by a high frequency of smoking, increased vascular stiffness, and the presence of lower extremity PAD [62,63]. It is likely that a high femoral plaque burden, even in the absence of lower extremity PAD symptoms or a reduced ABI, may be an integral part of these phenotypes. In turn, impaired ventricular–arterial coupling and an elevated Ea/Ees ratio may not only be components of these phenotypes, characterizing the systemic hemodynamic profile, but also key prognostic factors and potential therapeutic targets. One potential therapeutic implication is widespread screening for asymptomatic PAD in HF patients and early revascularization, including endovascular interventions with stent implantation and the application of innovative perivascular wraps [64,65,66]. Another promising approach is the expanded use of GLP1-RA in patients with CHF and extensive peripheral artery atherosclerosis, which may have benefits and modify the clinical course of both PAD and CHF [67,68].

The present study has several limitations: (1) The most important limitation is the limited sample size. According to the results of a post hoc analysis of the required sample size, it was found that 51 to 140 patients are sufficient for an accurate ROC analysis [37]. (2) A heterogeneous patient sample across a wide range of LVEF. Further research involving a larger cohort of patients is needed to allow for appropriate analysis within subgroups of patients with different LVEF. The heterogeneity of patients in relation to their clinical status is also an important limitation of the study. More than one-third of patients were stable outpatients with HFpEF receiving guideline-directed medical therapy. These patients likely contributed to the relatively low median NT-proBNP and a number of echocardiographic parameter values in the study cohort of patients. These findings are consistent with the results of several large-scale studies [69,70]. (3) The study design only makes it possible to determine associative relationships between studied indicators, and the results should not be interpreted as causal relationships. (4) The lack of vascular stiffness assessment, which would have allowed for an evaluation of the relationship between the impact of femoral plaque burden on cardiac remodeling and aortic stiffness. The most promising approach is to measure vascular stiffness by recording aortic pulse wave velocity (PWV), followed by calculating ventricular–arterial coupling as the ratio of PWV to GLS [32]. It is hypothesized that the use of PWV, considered the gold standard for assessment of vascular stiffness, and GLS, the gold standard for systolic dysfunction, will provide a more adequate and reproducible indicator of ventricular–arterial coupling that is less prone to variability associated with fluctuations in heart rate, blood pressure, and other factors [32]. However, this approach can be limited in patients with PAD undergoing stenting or graft implantation, as these devices have a direct effect on increasing PWV [71,72].

## 5. Conclusions

In patients with CHF aged 40–79 years, an increased femoral plaque burden was associated with reduced deformation and increased stiffness of the LA, as well as with reduced deformation and ejection fraction of the LV. Furthermore, the increased femoral plaque burden was associated with higher Ea, lower Ees, and an elevated Ea/Ees ratio. Ultrasound indicators of femoral plaque burden demonstrated moderate diagnostic performance in detecting a pathologically elevated Ea/Ees ratio.

## Figures and Tables

**Table 1:** Methodology of echocardiographic measurements.

Parameter	Measurement Method	References
Left Ventricular Myocardial Mass (LVMM)	Parasternal long-axis (PLAX) view in B-mode. M-mode measurements: left ventricular end-diastolic diameter (LVEDD), diastolic thickness of the posterior wall (PWT), and diastolic thickness of the interventricular septum (IVSd). LVMM = 0.8 × (1.04 × (((LVEDD/10) + (PWT/10) + (IVSd/10))^3^ − ((LVEDD/10))^3^)) + 0.6	[18,19,20]
Left Ventricular Mass Index (LVMI)	Body Surface Area (BSA) = √((height (cm) × weight (kg))/3600) LVMI =LVMM/Body Surface Area (BSA)	[18,19,20]
Left Atrial Volume (LAV)	LAV was measured in the apical four-chamber view in B-mode. Delineation of the left atrial endocardial borders during atrial diastole followed by volumetric analysis. It was performed similarly to the left ventricular volume measurement. Simpson’s method was applied for volume calculation in two orthogonal planes, excluding the pulmonary vein orifices and the left atrial appendage.	[18,19,20]
Left atrial volume index (LAVI)	LAVI = LAV/BSA	[18,19,20]
Left atrial-ventricular coupling index (LACI)	Left atrial end-diastolic volume (LAEDV)/Left ventricular end-diastolic volume (LVEDV)	[22,23]
Global Longitudinal Strain of the Left Atrium (LAGLS)	The maximum long-axis view of the left atrium was obtained in the apical four-chamber position. The endocardial border of the left atrium was then traced from one aspect of the mitral annulus to the opposite aspect, with linear extrapolation performed between the ostia of the left atrial appendage and the pulmonary veins.	[24,25]
Left Atrial Reservoir Strain (LASr)	It was measured as the difference between strain values at the time of mitral valve opening and at left ventricular end-diastole.	[24,26]
Left Atrial Stiffness Index (LASI)	(E/e′)/LASr	[27,28]
Global Longitudinal Strain of the Left Ventricle (LVGLS)	Measurements were performed during stable ECG recording of at least one complete cardiac cycle in the apical four-chamber, two-chamber, and long-axis views. Data analysis was performed in a semi-automated mode. The endocardial contour of the region of interest was traced manually in the apical views. After analysis of longitudinal strain from the three standard views, the software automatically produced a bull’s-eye plot, providing a topographic color-coded display of the calculated strain values for all 17 left ventricular segments.	[20,24]
Global Longitudinal Strain Rate of the Left Ventricle (LVGLSR)	The rate at which myocardial deformation occurs in one dimension.	[20,24]
Circumferential strain of the left ventricle (LVCS)	Measurements were performed during stable ECG recording of at least one cardiac cycle in the short-axis view of the left ventricle. Data analysis was performed in a semi-automated mode. The endocardial borders of the region of interest were delineated in the left ventricular short-axis views at the level of the mitral valve leaflets, papillary muscles, and left ventricular apex. After analysis of circumferential strain from the three standard views, the software automatically produced a bull’s-eye plot, providing a topographic color-coded display of the calculated strain values for all 17 left ventricular segments.	[20,24]
E/e′	Early diastolic transmitral flow velocity (E) (cm/s) Tissue Doppler Imaging: medial mitral annular early diastolic velocity (TDI E’ medial MV) Tissue Doppler Imaging: lateral mitral annular early diastolic velocity (TDI E’ lateral MV) E/e′ = E/((TDI E’ medial MV + TDI E’ lateral MV)/2) The ratio of early diastolic transmitral flow velocity (E) to early diastolic mitral annular velocity (e’). Tissue Doppler Imaging (TDI) was employed to measure the average mitral annular e’ velocity. The e’ velocity is measured in the lateral and septal basal segments in close proximity to the mitral annulus. Pulsed-wave Doppler was used to measure the early diastolic transmitral flow velocity from the apical four-chamber view. The sample volume was positioned parallel to the blood flow just distal to the mitral valve leaflet coaptation point on the left ventricular side, where transmitral flow velocity is maximal.	[20,29]
Ventricular elastance (Ees)	0.9 × Systolic Arterial Pressure (SAP)/Left Ventricular End-Systolic Volume (LVESV) Left ventricular end-systolic volume (LVESV) was measured in the apical four-chamber view.	[30,31]
Arterial elastance (Ea)	0.9 × SAP/Stroke Volume (SV) LVOT= Left Ventricular Outflow Tract VTI LVOT= Velocity − Time Integral of the Left Ventricular Outflow Tract SV= 0.785 × (diameter LVOT^2^) × VTI LVOT LVOT VTI was measured by placing the pulsed-wave (PW) Doppler sample volume in the left ventricular outflow tract just below the aortic valve and recording the flow velocity (cm/s). VTI LVOT, corresponding to the stroke distance (cm), was derived by planimetry of the Doppler spectral envelope. LVOT diameter was measured in mid-systole from the parasternal long-axis view in 2D mode. The measurement was taken 5–10 mm apical to the aortic valve using the inner-edge to inner-edge technique.	[30,31]
Left ventricular–arterial coupling	Ea/Ees	[30,31]

**Table 2:** Clinical and laboratory characteristics of the patients.

Characteristics	Patients (n = 89)
Male, n (%)/Female, n (%)	37 (41.6)/52 (58.4)
Age, Me (IQR)	73.0 (65.7; 75.0)
HFrEF, n (%)	26 (29.2)
HFmrEF, n (%)	12 (13.5)
HFpEF, n (%)	51 (57.3)
NYHA	
I, n (%)	3 (3.40)
II, n (%)	18 (20.2)
III, n (%)	57 (64.0)
IV, n (%)	11 (12.4)
BMI, kg/m^2^, Me (IQR)	27.1 (24.1; 31.7)
Obesity, n (%)	29 (32.6)
Current smoking, n (%)	21 (23.6)
Hypertension, n (%)	89 (100.0)
CAD, n (%)	39 (43.8)
Coronary revascularization, n (%)	9 (10.1)
Type 2 diabetes mellitus, n (%)	24 (27.0)
Atrial fibrillation, n (%)	15 (16.9)
Antiplatelet therapy, n (%)	25 (28.1)
Beta-blockers, n (%)	61 (68.5)
RAAS inhibitors, n (%)	76 (85.4)
SGLT2 inhibitors, n (%)	41 (46.1)
MRAs, n (%)	53 (59.6)
Diuretics, n (%)	39 (43.8)
Statins, n (%)	71 (79.8)
NT-proBNP, pg/mL, Me (IQR)	214.9 (14.0; 1478.0)
hs-CRP, mg/L, Me (IQR)	4.02 (1.08; 9.73)
TC, mmol/L, Me (IQR)	4.69 (3.97; 5.81)
LDL-C, mmol/L, Me (IQR)	2.74 (2.28; 3.59)
HDL-C, mmol/L, Me (IQR)	1.14 (0.96; 1.34)
Non-HDL-C, mmol/L, Me (IQR)	3.58 (2.92; 4.58)
TG, mmol/L, Me (IQR)	1.09 (0.79; 1.89)
Glucose, mmol/L, Me (IQR)	6.22 (5.22; 7.99)
Creatinine, μmol/L, Me (IQR)	95.0 (81.0; 117.5)
eGFR, mL/min/1.73 m^2^, Me (IQR)	55.2 (46.7; 68.7)
Potassium, mmol/L, Me (IQR)	4.30 (3.95; 4.80)
Sodium, mmol/L, Me (IQR)	138.0 (135.0; 141.0)

**Table 3:** Results of lower extremity arterial duplex ultrasound and echocardiography.

Characteristics	Patients (n = 89)
Atherosclerotic plaques in lower extremity arteries, n (%)	81 (91.0)
Number of atherosclerotic plaques in lower extremity arteries (TPN), Me (IQR)	3.00 (2.00; 4.00)
Total plaque area in femoral arteries (fTPA), mm^2^, Me (IQR)	57.0 (35.5; 86.5)
Total plaque thickness in femoral arteries (fTPT), mm, Me (IQR)	4.98 (3.51; 7.74)
MaxStenosis of Lower Extremity Arteries, %, Me (IQR)	28.0 (21.0; 38.0)
ABIm, Me (IQR)	1.19 (1.10; 1.30)
ABIm < 0.9, n (%)	7 (7.86)
IVSd, mm, Me (IQR)	10.0 (9.00; 12.0)
PWT, mm, Me (IQR)	10.0 (9.00; 11.0)
LVMM, g, Me (IQR)	206.0 (167.8; 265.0)
LVMI, g/m^2^, Me (IQR)	108.5 (81.7; 143.5)
LAV, mL, Me (IQR)	65.5 (53.5; 87.5)
LAVI, mL/m^2^, Me (IQR)	36.0 (30.0; 45.0)
LVEDV, mL, Me (IQR)	93.5 (72.0; 116.5)
LVESV, mL, Me (IQR)	43.0 (30.0; 63.5)
LACI, Me (IQR)	0.77 (0.56; 0.98)
LAGLS, %, Me (IQR)	18.1 (11.2; 24.9)
LASr, %, Me (IQR)	15.5 (9.65; 21.0)
LASI, Me (IQR)	0.41 (0.24; 0.70)
LVEF, %, Me (IQR)	52.7 (38.7; 61.5)
LVGLS, %, Me (IQR)	−9.20 (−11.2; −5.75)
Reduced LVGLS, n (%)	76 (85.4)
LVSR, s^−1^, Me (IQR)	−0.25 (−0.60; 0.60)
LVCS, %, Me (IQR)	−12.2 (−17.6; −8.10)
E/e′, Me (IQR)	6.00 (4.15; 7.80)
Ees, mmHg/mL, Me (IQR)	2.68 (1.78; 4.03)
Ea, mmHg/mL, Me (IQR)	1.87 (1.33; 2.76)
Ea/Ees, Me (IQR)	0.67 (0.39; 1.18)
Ea/Ees > 1.0, n (%)	35 (39.3)

**Table 4:** Results of logistic regression analysis.

Indicator	Model 1		Model 2		Model 3	
	OR (95% CI)	*p*	OR (95% CI)	*p*	OR (95% CI)	*p*
fTPA > 45.0 mm^2^	3.69 (1.47; 9.25)	0.005	2.10 (0.72; 6.13)	0.175	3.38 (0.75; 14.9)	0.114
fTPT > 5.26 mm	2.83 (1.16; 6.89)	0.022	1.62 (0.54; 4.83)	0.387	3.76 (0.87; 16.2)	0.076
TPN > 3.00	2.38 (0.98; 5.78)	0.055	1.25 (0.41; 3.79)	0.697	1.67 (0.41; 6.85)	0.478
MaxStenosis > 38.0%	8.00 (2.35; 27.2)	0.001	4.49 (1.10; 18.2)	0.036	8.44 (1.50; 47.4)	0.015

Model 1: unadjusted; Model 2: adjustment for age, sex and LVEF, Model 3: adjustment for age, sex, BMI, CAD, T2DM, antiplatelet therapy, beta-blockers, RAAS inhibitors, MRAs, diuretics, statins, creatinine, total cholesterol, NT-proBNP; OR: odds ratio.

## Data Availability

The data used to support the findings of this study are available from the corresponding author upon reasonable request.
